# Complications of Interventional Versus Surgical Closure of Patent Ductus Arteriosus in Very Preterm Infants—A Retrospective Analysis

**DOI:** 10.3390/jcdd13010022

**Published:** 2025-12-31

**Authors:** Karla Girke, Christoph Bührer, Bernd Opgen-Rhein, Boris Metze, Christoph Czernik

**Affiliations:** 1 Department of Neonatology, Charité—Universitätsmedizin Berlin, 10117 Berlin, Germany; karla.girke@charite.de (K.G.); boris.metze@charite.de (B.M.); christoph.czernik@charite.de (C.C.); 2 Department of Congenital Heart Disease—Pediatric Cardiology, Deutsches Herzzentrum der Charité, Charité—Universitätsmedizin Berlin, 13353 Berlin, Germany; bernd.opgen-rhein@dhzc-charite.de

**Keywords:** patent ductus arteriosus (PDA), very low birth weight infants, preterm neonates, transcatheter closure, surgical ligation, complications, mechanical ventilation, necrotizing enterocolitis (NEC)

## Abstract

Introduction. Patent ductus arteriosus (PDA) is the most common cardiac anomaly in preterm newborns and may aggravate respiratory disease. Invasive closure options after failure of medical treatment include surgical ligation (SL) and transcatheter closure (TCC). Reports on side effects of intravenous contrast media are scarce. Methods. In this retrospective single-center study, we compared 35 preterm infants below 1500 g birth weight undergoing SL with 35 matched infants undergoing TCC. Outcomes were procedural success, complications and postprocedural ventilation. Results. Closure success was high in both groups (97% SL vs. 86% TCC, *p* = 0.106). One SL patient underwent re-operation after accidental clipping of the left pulmonary artery, and eight patients (24%) had endoscopy-diagnosed vocal cord palsy after SL. Six TCC patients had complications that required further action, including device embolization, device failure and one case of late device migration that resulted in aortic arch obstruction requiring intervention, and 4 TCC patients developed necrotizing enterocolitis (NEC)-like disease within 24 h, requiring surgery in one patient. SL was associated with longer duration of mechanical ventilation (24 h vs. 144 h, *p* < 0.001), as opposed to TCC, and higher rates of bronchopulmonary dysplasia (86% vs. 53%, *p* = 0.004). Discussion. Both techniques achieve high success but differ in complication profiles. TCC may reduce respiratory morbidity. NEC-like disease (probably linked to intravenous administration of contrast agents) warrants further investigation.

## 1. Introduction

Patent ductus arteriosus (PDA), defined as an arterial duct that remains open after the third day of life in a newborn [1], affects up to 80% of preterm born infants, depending on their gestational age and birthweight [2]. While most PDAs do not require medical treatment and spontaneously close in the preterm infant’s first week of life [3,4,5], a PDA that is defined as hemodynamically significant (hsPDA) often calls for medical action.

Inhibition of prostaglandin synthesis by indomethacin, ibuprofen, or acetaminophen during the first two weeks of life may result in PDA closure [6,7,8]. However, pharmacological treatment during the first week of life may be associated with more harm than benefit, including increased rates of death and bronchopulmonary dysplasia [9,10,11]. There is a high rate of spontaneous closure while prospects of successful pharmacological closure dwindle during the second and third week of life [12,13]. If medical treatment fails and infants cannot be weaned off mechanical ventilation after 10–14 days [14], rescue therapy by surgical ligation of the ductus and transcatheter percutaneous closure will be discussed. Surgical ligation can be performed at the bedside and has a near 100% success rate. However, there are several possible complications, including left-sided vocal cord paralysis by recurrent laryngeal nerve injury, prolonged post-operative ventilator-dependency and high opioid need due to the painful procedure. Chylothorax, pneumothorax, accidental clipping of the left pulmonary artery, and death are further possible perioperative adverse events [15,16,17,18].

Following the general trend in cardiovascular medicine with increasingly more transcatheter interventions and less surgical procedures [19,20], a novel method to close the PDA via transcatheter approach in even the smallest preterm infants has emerged over the past 5 years after FDA approval of a closure device for infants with a minimal weight of 700 g [21]. Transcatheter closure is a minimally invasive and less painful approach, associated with fewer complications than surgery while being close to equally effective [17,22,23,24]. Furthermore, comparison of the two methods has shown similar neurodevelopmental outcomes [25,26] and no difference in respiratory outcomes despite shorter ventilation duration [27,28], but higher survival rates and shorter lengths of hospital stay after TCC [23]. Nevertheless, besides radiation and radiocontrast agent exposure, a number of adverse events have been reported including vessel stenosis by a protruding device, device embolization, vessel- or myocardial perforation, and death [29]. The procedure exposes infants to the risk of hypothermia during transfer from the neonatal ICU to the catheterization laboratory but currently few institutions perform the procedure at the bedside [30].

A recently published practice guideline by Ambalavanan et al. recommends an individual and multidisciplinary approach when deciding whether transcatheter or surgical closure are favorable for a patient but highlights the need for further studies regarding the potential risks and long-term results of transcatheter closure [1].

Consequently, given the limited data comparing both approaches in preterm infants and the potential risks associated with each technique, we conducted a monocentric, retrospective case–control-study to analyze outcomes and complications of percutaneous and surgical PDA closure in preterm children with a birthweight below 1500 g.

## 2. Methods

### 2.1. Study Design and Population

This retrospective, single-center study was conducted at Charité—Universitätsmedizin Berlin. All preterm infants below 1500 g birthweight who underwent catheter-based PDA closure between 2019 and 2023 were included. From a cohort of 52 eligible surgically treated patients (2013–2023), a matched cohort was selected based on birth weight, gestational age, and sex. The study was approved by the Charité ethical committee and the board of study registration (EA2/024/24).

All infants received echocardiographic screening on postnatal days 5 to 7 in search for PDA and to exclude duct-dependent congenital heart disease. Hemodynamic significance was evaluated using echocardiographic criteria (e.g., ductal diameter > 2 mm, left atrial enlargement) and clinical parameters (e.g., FiO_2_ > 0.3, persistent ventilation-dependency). First-line treatment in all patients consisted of ibuprofen and/or paracetamol.

Percutaneous closure was performed in the catheter laboratory under sedation and analgesia, when possible. Devices used were Amplatzer Duct Occluder (ADO) IIas or Amplatzer Piccolo Occluder depending on size and configuration of the duct as proposed by Sathanandam et al. 2020 [21]. Imaging of the duct was conducted using fluoroscopy and intravenous contrast agent (Imeron^®^ 300, Bracco Imaging Deutschland GmbH, Konstanz, Germany). Surgical ligation was performed under general anesthesia by the cardiothoracic surgery department via posterior or left-lateral thoracotomy. After successful PDA-closure, all patients received the same standard feeding protocol.

### 2.2. Data Collection and Statistical Analysis

Demographic, procedural and outcome data were extracted from both electronic and paper-based records. Parameters related to complications, procedural success, and respiratory support were recorded. Post-procedural assessment included screening of discharge letters and nurses’ and doctor’s notes for gastrointestinal symptoms and abnormalities as well as any other possible complications detected after transcatheter closure and surgical ligation.

Data analysis was performed using IBM SPSS Statistics version 31 (IBM Corporation, Armonk, NY, USA). Categorical variables were compared using chi-square tests; continuous variables using Mann–Whitney U tests. A significance level of *p* < 0.05 was used.

### 2.3. Outcomes

Primary outcomes were procedural success as well as frequency and types of complications after transcatheter versus surgical PDA-closure. Secondary outcomes included postprocedural ventilation duration. Ventilation status was assessed based on the timing of endotracheal intubation, differentiating between pre-existing intubation and intubation performed specifically for the procedure and patients were grouped accordingly. In the setting of complication rates, we focused on gastrointestinal complications which we defined as necrotizing enterocolitis (NEC) or NEC-like symptoms (such as distended or tender abdomen, hematochezia, and ileus) after intervention or surgery. We also assessed if patients showed a post-ligation cardiac syndrome (PLCS), which was defined as relevant post-procedural hemodynamic instability with need for catecholamines and/or intensification of ventilator settings as described by Giesinger et al. [31]. Vocal cord paralysis following surgery was recorded if the diagnosis was confirmed by laryngoscopy.

## 3. Results

### 3.1. Baseline Characteristics

A total of 70 preterm infants were included (35 surgical ligations, 35 transcatheter closures). Groups were comparable in gestational age, birth weight, and sex distribution. At the time of intervention, however, infants in the transcatheter group were significantly older and heavier (median 47 days, 1495 g) compared to the surgical group (25 days, 1015 g, *p* < 0.001). Baseline characteristics of the study groups are summarized in Table 1.

### 3.2. Procedures and Complications

The procedures differed significantly regarding duration, performance at the bedside, respiratory management and in the parameters regarding the specific techniques (such as radiation and contrast agent doses and access). One patient underwent transcatheter closure again after the first failed attempt, which is why the data contains 36 interventions for 35 patients.

All patients in the surgical group were intubated at the time of the procedure, compared to only 63.9% in the interventional group (*p* < 0.001). Of those, 11 (31.4%) surgically treated patients were intubated directly prior to the procedure, while 24 (68.6%) had been intubated before due to the patient’s general status. Of those ventilated in the transcatheter group, 16 (44.4%) patients had been intubated earlier than directly prior to their procedure. Median total procedure time (including anesthetic management) was significantly longer for surgery (85 vs. 66 min, *p* = 0.006), as was pure procedural time (45 vs. 26 min, *p* < 0.001). Surgical procedures were almost exclusively performed at the bedside (91%), whereas interventional procedures were performed exclusively in the catheterization lab. As expected, catheter-based interventions involved exposure to radiation and iodinated intravenous contrast agents (ICA) (median 2.7 mL/kg). Detailed procedural parameters are summarized in Table 2.

Both procedures demonstrated high success rates (97% surgical vs. 86% interventional, *p* = 0.106). No life-threatening or deadly complications occurred in either group. While overall complication rates did not differ significantly between the groups (SL 43% vs. TCC 48%, *p* = 0.64), BPD occurred significantly more often in the surgical group (86% vs. 53%, *p* = 0.004). The most common complication in both groups was PLCS (SL: 34% vs. TCC: 29%, *p* = 0.792). Vocal cord paralysis confirmed by laryngoscopy occurred exclusively after surgery in 8 (24%) surgically treated patients.

Post-procedural acute gastrointestinal complications resembling NEC occurred exclusively after transcatheter intervention (13% vs. 0%, *p* = 0.044), as shown in Table 3. These included one case of established NEC which required surgical treatment and three cases of NEC-like disease with post-procedural hematochezia and/or ileus. The symptoms developed within 24 h in all four intervention patients. Table 4 shows these cases and their symptoms in more detail. The median ICA volume per kilogram of body weight was significantly higher in infants who developed gastrointestinal symptoms compared to those who did not (3.11 mL/kg vs. 2.17 mL/kg, *p* = 0.012).

In the interventional group, 5 patients had complications that required (re-)intervention, including device embolization, device failure and one case of late device migration with resulting aortic arch obstruction. One patient in the surgical group underwent re-operation after accidental clipping of the left pulmonary artery. Eight patients showed mild aortic or pulmonary artery stenosis with mildly elevated Doppler flow velocity after interventional closure. In these patients, follow-up echocardiography performed between 3 weeks and 4 months after the intervention showed no progression or need for further therapy.

### 3.3. Respiratory Outcomes

Postprocedural ventilation duration was significantly shorter in the interventional group (median 24 h vs. 144 h, *p* < 0.001). Subgroup analysis based on pre-existing ventilation status revealed significantly shorter median ventilation duration after transcatheter closure in patients already intubated (120 h vs. 192 h, *p* = 0.026).

## 4. Discussion

This study aimed to compare surgical and transcatheter PDA closure in very low birth weight preterm infants.

Our data show that both procedures are similarly effective regarding closure success but differ in respiratory outcomes. Surgical PDA closure was more often associated with complications such as recurrent nerve paresis, while complications regarding the correct placement of the device, including embolization and vessel stenosis, were observed after interventional closure. Most minor complications in the interventional group did not require further treatment. Post-ligation cardiac syndrome was observed with similar frequency in both groups and limited to less than one week.

**Figure 1:**
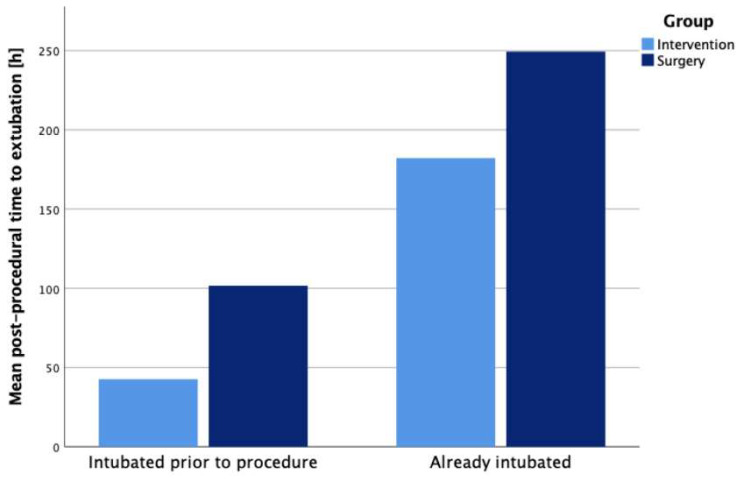
Mean post-procedural ventilation time over pre-existing ventilation status.

Ventilation duration after the procedure was significantly shorter in the transcatheter group throughout all subgroups, but especially among already intubated patients, as demonstrated in Figure 1. Infants in the interventional group showed a lower rate of BPD compared to the surgical group despite being older, which possibly indicates that increased age alone may not be associated with a higher risk for PDA-related comorbidities.

NEC or NEC-like disease was exclusively observed after percutaneous closure. Percutaneous PDA closure is one of the very few occasions where a preterm newborn is subject to intravenous contrast agent administration. We hypothesize that, similar to adult red blood cell transfusion administration in preterm infants [32,33], ICA negatively affects intestinal blood perfusion secondary to high viscosity [34]. The association between GI symptoms and ICA has been described in adult patients in whom nausea and vomiting are recognized adverse events of ICA administration [35,36]. Bowel wall swelling has been observed after ICA, resulting in nausea, vomiting, and mild diarrhea [37,38]. While the median dosage of ICA (2.7 mL/kg body weight) was similar to that reported elsewhere [21,39], infants who developed NEC-like disease had received significantly higher doses of ICA per kilogram body weight than those without abdominal symptoms. Characteristics of patients that showed gastrointestinal symptoms after their PDA closure are shown in Table 5. While symptoms and severity differed, all patients had markedly elevated CRP serum concentrations and one also showed thrombocytopenia within 72 h after intervention. Notably, all NEC(-like)-cases displayed symptoms immediately or within 24 h after TCC. These findings warrant further investigation but may call for cautious feeding protocols post procedure. However, we would like to emphasize that a possible causal connection between NEC-like symptoms and ICA use is hypothetical and has not been subject of previous reports.

The main limitations of this study include its retrospective design, monocentric setting, and limited sample size. Differentiation between causal links or bias due to the small group sizes remains difficult. Furthermore, despite being matched for birth weight and gestational age, the interventional group had a higher body weight at the time of PDA closure, reflecting both later procedural timing and case selection. This difference may have contributed to the lower incidence of complications and shorter ventilation durations observed in this group. Moreover, the two treatment arms span different time periods, which may have introduced bias despite matching. Nonetheless, our matched cohort design and thorough screening for and reporting of complications strengthen the interpretability and clinical relevance of our results.

Our findings align with previously published studies suggesting comparable efficacy between both closure methods, with differing complication profiles [22,42]. The success rates observed in our study were slightly lower compared to larger studies [21], possibly reflecting a continuing learning curve due to limited case experience and the novelty of the procedure. The significantly shorter ventilation durations observed after interventional closure in our cohort are consistent with recent retrospective analyses [24,43]. However, this difference may largely reflect the older age and higher body weight of the percutaneously treated infants; thus, a causal relationship between the type of procedure and reduced ventilation time cannot be established from these data alone. A recent study investigating respiratory outcomes following PDA closure showed no difference between percutaneous and surgical approaches after adjustment for birth year and maturity [27], which might be a more accurate assessment of this aspect. The higher BPD-rates in our surgical cohort could be attributed to the earlier timing of surgery as shown by Clyman et al. [44].

As of October 2025, there is no published evidence supporting our thesis of a connection between ICA in preterm newborns and NEC. However, we deemed it important to note this possible connection with gastrointestinal symptoms with established NEC and the use of ICA in preterm VLBW newborns. Exclusively echocardiography-led transcatheter PDA closure, as described by Georgiev et al. and others [45,46,47], allows for bedside procedures that avoid radiation exposure and eliminates the need for ICA. Considering our findings regarding the possible association between ICA and NEC, the relevance of this novel method gains further significance for vulnerable preterm patients.

## 5. Conclusions

In very low birth weight infants undergoing PDA closure, both surgical and interventional approaches demonstrated high success rates, complication profiles differed, and ventilation durations were shorter following interventional closure. NEC or NEC-like gastrointestinal symptoms were observed after interventional closure only, possibly related to the use of ICA media. These findings support the use of interventional PDA closure as a minimally invasive rescue therapy in older preterm infants with ibuprofen-resistant PDA. The potential risk of NEC by ICA warrants further investigation by prospective studies and highlights the growing importance of echo-guided transcatheter PDA closure as a viable and potentially safer alternative.

## Figures and Tables

**Table 1:** Study group characteristics.

Parameter	Surgery (*n* = 35)	Intervention (*n* = 35)	*p*-Value
Gestational age (weeks)	25.1 (23.2–31.6)	25.1 (22.0–33.5)	0.622
Birth weight (g)	740 (440–1220)	745 (445–1285)	0.381
Female	19 (54)	18 (51)	0.999
5-min-Apgar score	7 (2–9)	7 (3–9)	0.603
Day of life (DOL) at procedure (d)	25 (14–52)	47 (13–286)	<0.001
Weight at procedure (g) (range)	1015 (700–1905)	1495 (860–5500)	<0.001

Data are median (range) or *n* (%).

**Table 2:** Procedural characteristics.

Parameter	Surgery	Intervention	*p*-Value
*n*	35	36	
Intubated	35 (100)	23 (63.9)	<0.001
Total Procedure Time (min)	85 (45–285)	66 (50–237)	0.006
Procedural Time (min)	45 (10–104)	26 (16–187)	<0.001
Bedside/NICU Procedure	32 (91.4)	0 (0)	<0.001
Contrast Agent per kg (mL/kg)	—	2.7 (0.91–6.45)	<0.001
Fluoroscopy Time (min)	—	3 (1.4–81.9)	<0.001
DAP per kg (cGy*cm^2^/kg)	—	4.13 (1.32–39.52)	<0.001
Arterial Access via A. femoralis	—	5 (13.9)	<0.001
Venous Access via V. femoralis	—	31 (86.1)	<0.001
Posterior Thoracotomy (%)	24 (68.6)	—	<0.001
Lateral Thoracotomy (%)	9 (25.7)	—	<0.001
Body temperature post-procedure (°C)	37.0 (35.1–39.4)	37.0 (35.4–39.1)	0.948

Data are median (range) or *n* (%).

**Table 3:** PDA-associated conditions.

Condition	Surgery	Intervention	*p*-Value
Bronchopulmonary Dysplasia (O_2_ requirement at 36 weeks postmenstrual age)	30 (85.7)	19 (52.8)	0.004
Vocal cord palsy	8 (24.2)	0 (0.0)	0.002
Mild LPA/Ao stenosis	0 (0.0)	8 (22.2)	0.001
Acute NEC-like disease	0 (0.0)	4 (12.9)	0.044

Data are *n* (%).

**Table 4:** Complications.

Case No	Sex	Birth Weight (g)	Gestational Age (Weeks)	PDA Treatment	Complication
8	M	770	26 + 0	Intervention	Device embolization with LPA obstruction, unproblematic explanation of device
12	F	590	23 + 3	Intervention	Late device migration and percutaneous balloon angioplasty of the aortic isthmus 5 months after initial procedure
15	M	445	22 + 0	Intervention	Device failure (duct too large)
17	M	1240	29 + 1	Intervention	Device embolization in right lung, surgical retrieval
21	F	855	25 + 1	Intervention	Device failure (duct too large)
24	M	990	28 + 6	Intervention	Embolization in MPA, unproblematic retrieval
36	M	550	23 + 2	Surgery	Left laryngeal nerve paresis
40	F	470	25 + 6	Surgery	Left laryngeal nerve paresis
41	M	885	27 + 2	Surgery	Accidental LPA clipping, need for corrective surgery
45	F	660	24 + 0	Surgery	Left laryngeal nerve paresis
46	M	650	23 + 3	Surgery	Left laryngeal nerve paresis
53	F	735	25 + 4	Surgery	Left laryngeal nerve paresis
56	F	835	24 + 4	Surgery	Left laryngeal nerve paresis
66	F	702	25 + 3	Surgery	Left laryngeal nerve paresis
68	F	487	23 + 5	Surgery	Left laryngeal nerve paresis

**Table 5:** Acute gastrointestinal abnormalities after PDA closure.

Case No	9	10	16	19
PDA treatment	Intervention	Intervention	Intervention	Intervention
Time of onset of symptoms after procedure	24 hours	24 hours	24 hours	12 hours
Modified Bell’s stage ^1^	1a	1b	1b	2a
Hematochezia		yes	yes	yes
Ileus	yes			yes
Pneumatosis intestinalis				yes
Peak C-reactive protein within 72 h after procedure [mg/L]	36.2	13.2	69.3	188.7
Peak IL-6 within 72 h after procedure (ng/L)	443	50.6	>50,000	3083
Lowest platelet counts within 72 h after procedure [/nL]	164	392	52	174
Surgery				yes

^1^ As described by Bell et al. (1978) and modified by Walsh and Kliegman et al. (1986) [40,41]

## Data Availability

The data presented in this study are available on request from the corresponding author.

## References

[1] Ambalavanan N., Aucott S.W., Salavitabar A., Levy V.Y. (2025). Patent Ductus Arteriosus in Preterm Infants. Pediatrics.

[2] Sung S.I., Chang Y.S., Kim J., Choi J.H., Ahn S.Y., Park W.S. (2019). Natural evolution of ductus arteriosus with noninterventional conservative management in extremely preterm infants born at 23–28 weeks of gestation. PLoS ONE.

[3] de Klerk J.C.A., Engbers A.G.J., van Beek F., Flint R.B., Reiss I.K.M., Völler S., Simons S.H.P. (2020). Spontaneous Closure of the Ductus Arteriosus in Preterm Infants: A Systematic Review. Front. Pediatr..

[4] He M., Yang Z., Gan T., Tang J., Ran S., Zhang K. (2023). Echocardiographic parameters predicting spontaneous closure of ductus arteriosus in preterm infants. Front. Pediatr..

[5] Semberova J., Sirc J., Miletin J., Kucera J., Berka I., Sebkova S., O’Sullivan S., Franklin O., Stranak Z. (2017). Spontaneous Closure of Patent Ductus Arteriosus in Infants = 1500 g. Pediatrics.

[6] Hundscheid T., van den Broek M., van der Lee R., de Boode W.P. (2019). Understanding the pathobiology in patent ductus arteriosus in prematurity-beyond prostaglandins and oxygen. Pediatr. Res..

[7] Jasani B., Mitra S., Shah P.S. (2022). Paracetamol (acetaminophen) for patent ductus arteriosus in preterm or low birth weight infants. Cochrane Database Syst. Rev..

[8] Engbers A.G.J., Völler S., Flint R.B., Goulooze S.C., de Klerk J., Krekels E.H.J., van Dijk M., Willemsen S.P., Reiss I.K.M., Knibbe C.A.J. (2022). The Effect of Ibuprofen Exposure and Patient Characteristics on the Closure of the Patent Ductus Arteriosus in Preterm Infants. Clin. Pharmacol. Ther..

[9] Buvaneswarran S., Wong Y.L., Liang S., Quek S.C., Lee J. (2025). Active Treatment vs Expectant Management of Patent Ductus Arteriosus in Preterm Infants: A Meta-Analysis. JAMA Pediatr..

[10] Mitra S., Scrivens A., Fiander M., Disher T., Weisz D.E. (2025). Early treatment versus expectant management of hemodynamically significant patent ductus arteriosus for preterm infants. Cochrane Database Syst. Rev..

[11] Laughon M.M., Thomas S.M., Watterberg K.L., Kennedy K.A., Keszler M., Ambalavanan N., Davis A.S., Slaughter J.L., Guillet R., Colaizy T.T. (2025). Expectant Management vs Medication for Patent Ductus Arteriosus in Preterm Infants: The PDA Randomized Clinical Trial. JAMA.

[12] Dani C., Sassudelli G., Milocchi C., Vangi V., Pratesi S., Poggi C., Corsini I. (2025). Effectiveness of repeated pharmacological courses for patent ductus arteriosus in preterm infants. Early Hum. Dev..

[13] Sharma P., Gearhart A., Beam K., Spyropoulos F., Powell A.J., Beam A., Levy P. (2024). Perinatal Factors Associated with Successful Pharmacologic Closure of the Patent Ductus Arteriosus in Premature Infants. Pediatr. Cardiol..

[14] Mitra S., Bischoff A.R., Sathanandam S., Lakshminrusimha S., McNamara P.J. (2024). Procedural closure of the patent ductus arteriosus in preterm infants: A clinical practice guideline. J. Perinatol..

[15] Ashfaq A., Rettig R.L., Chong A., Sydorak R. (2022). Outcomes of patent ductus arteriosus ligation in very low birth weight premature infants: A retrospective cohort analysis. J. Pediatr. Surg..

[16] Avsar M.K., Demir T., Celiksular C., Zeybek C. (2016). Bedside PDA ligation in premature infants less than 28 weeks and 1000 grams. J. Cardiothorac. Surg..

[17] Saini A., Hamrick S., Adamson M., Bhombal S., Hash S., Kim D.W., LeFevre A.S., Long J., Mills M., Ligon R.A. (2025). Impact of PDA Closure Methodology on Peri-procedural Opioid Utilization in Preterm Neonates, a Cross-Sectional Review in a Tertiary Pediatric Healthcare System. Pediatr. Cardiol..

[18] Wei Y.J., Ju Y.T., Hsieh M.L., Kan C.D., Lin Y.C., Wang J.N. (2023). Surgical ligation, not transcatheter closure, associated with a higher severity of bronchopulmonary dysplasia in extremely preterm infant intervened for patent ductus arteriosus. Pediatr. Pulmonol..

[19] Mitchell C.C., Rivera B.K., Cooper J.N., Smith C.V., Berman D.P., Slaughter J.L., Backes C.H. (2019). Percutaneous closure of the patent ductus arteriosus: Opportunities moving forward. Congenit. Heart Dis..

[20] Shah Z.S., Clark R.H., Patt H.A., Backes C.H., Tolia V.N. (2023). Trends in Procedural Closure of the Patent Ductus Arteriosus among Infants Born at 22 to 30 Weeks’ Gestation. J. Pediatr..

[21] Sathanandam S.K., Gutfinger D., O’Brien L., Forbes T.J., Gillespie M.J., Berman D.P., Armstrong A.K., Shahanavaz S., Jones T.K., Morray B.H. (2020). Amplatzer Piccolo Occluder clinical trial for percutaneous closure of the patent ductus arteriosus in patients ≥700 grams. Catheter. Cardiovasc. Interv..

[22] Forero-Florez S.C., Ball M.A.Z., Escobar-Díaz M.C., Sanchez-de-Toledo J., Carretero J., Camprubí-Camprubí M. (2024). Percutaneous versus surgical closure of patent ductus arteriosus in low-weight premature infants: 10-year experience in a tertiary center. An. Pediatr. (Engl. Ed.).

[23] Leahy B.F., Edwards E.M., Ehret D.E.Y., Soll R.F., Yeager S.B., Flyer J.N. (2024). Transcatheter and Surgical Ductus Arteriosus Closure in Very Low Birth Weight Infants: 2018–2022. Pediatrics.

[24] Regan W., Benbrik N., Sharma S.-R., Auriau J., Bouvaist H., Bautista-Rodriguez C., Sirico D., Aw T.-C., di Salvo G., Foldvari S. (2020). Improved ventilation in premature babies after transcatheter versus surgical closure of patent ductus arteriosus. Int. J. Cardiol..

[25] Fernandez M.C., Kase J.S., Giamelli J., Reichlin A. (2024). Morbidity and neurodevelopmental outcomes at 2 years in preterm infants undergoing percutaneous transcatheter closure vs. surgical ligation of the PDA. J. Perinatol..

[26] Kaluarachchi D.C., Chock V.Y., Do B.T., Rysavy M.A., Sankar M.N., Laughon M.M., Backes C.H., Colaizy T.T., Bell E.F., McNamara P.J. (2025). Comparison of neurodevelopmental outcomes of extremely preterm infants undergoing trans-catheter closure of the patent ductus arteriosus compared to surgical ligation. J. Perinatol..

[27] Chock V.Y., Bhombal S., Davis A.S., Sankar M.N., Do B.T., Laughon M.M., Van Meurs K.P., Backes C.H., McNamara P.J. (2025). Respiratory Outcomes After Transcatheter vs Surgical Patent Ductus Arteriosus Closure in Preterm Infants. JAMA Netw. Open.

[28] Tabb C., Aggarwal S., Bajaj M., Natarajan G. (2023). Comparative Effectiveness of Surgical Ligation and Catheter Closure of Patent Ductus Arteriosus in Preterm Infants. Pediatr. Cardiol..

[29] Bischoff A.R., Jasani B., Sathanandam S.K., Backes C., Weisz D.E., McNamara P.J. (2021). Percutaneous Closure of Patent Ductus Arteriosus in Infants 1.5 kg or Less: A Meta-Analysis. J. Pediatr..

[30] Aw T.C., Chan B., Singh Y. (2023). Transport and Anaesthesia Consideration for Transcatheter Patent Ductus Arteriosus Closure in Premature Infants. J. Cardiovasc. Dev. Dis..

[31] Giesinger R.E., Bischoff A.R., McNamara P.J. (2019). Anticipatory perioperative management for patent ductus arteriosus surgery: Understanding postligation cardiac syndrome. Congenit. Heart Dis..

[32] Dang D., Gu X., Jiang S., Li W., Zhou W., Cao Y., Lee S.K., Wu H., Zhou J. (2024). RBC transfusion and necrotizing enterocolitis in very preterm infants: A multicenter observational study. Sci. Rep..

[33] Bellach L., Eigenschink M., Hassanein A., Savran D., Salzer U., Müllner E.W., Repa A., Klebermass-Schrehof K., Wisgrill L., Giordano V. (2022). Packed red blood cell transfusion in preterm infants. Lancet Haematol..

[34] Kok M., Mihl C., Mingels A.A., Kietselaer B.L., Mühlenbruch G., Seehofnerova A., Wildberger J.E., Das M. (2014). Influence of contrast media viscosity and temperature on injection pressure in computed tomographic angiography: A phantom study. Invest. Radiol..

[35] Ha J.Y., Choi Y.H., Cho Y.J., Lee S., Lee S.B., Choi G., Cheon J.E., Kim W.S. (2020). Incidence and Risk Factors of Nausea and Vomiting after Exposure to Low-Osmolality Iodinated Contrast Media in Children: A Focus on Preparative Fasting. Korean J. Radiol..

[36] Neeman Z., Abu Ata M., Touma E., Saliba W., Barnett-Griness O., Gralnek I.M., Rock W., Bisharat N. (2021). Is fasting still necessary prior to contrast-enhanced computed tomography? A randomized clinical study. Eur. Radiol..

[37] Hu X.H., Gong X.Y., Hu P. (2012). Transient small bowel angioedema due to intravenous iodinated contrast media. World J. Gastroenterol..

[38] Ishii S., Yamakuni R., Tsuchiya T., Yamaki A., Hara J., Sugawara S., Sekino H., Fukushima K., Ito H. (2024). Incidence of Bowel Wall Swelling Induced by Iodine-Contrast Media and Its Association With Gastrointestinal Manifestation. J. Comput. Assist. Tomogr..

[39] Herron C., Forbes T.J., Kobayashi D. (2022). Renal Function After Transcatheter Piccolo Patent Ductus Arteriosus Closure With Contrast Angiography in Extremely Premature Infants. Am. J. Cardiol..

[40] Bell M.J., Ternberg J.L., Feigin R.D., Keating J.P., Marshall R., Barton L., Brotherton T. (1978). Neonatal necrotizing enterocolitis. Therapeutic decisions based upon clinical staging. Ann. Surg..

[41] Walsh M.C., Kliegman R.M. (1986). Necrotizing enterocolitis: Treatment based on staging criteria. Pediat. Clin. N. Am..

[42] Melchior C.D.S., Neves G.R., de Oliveira B.L., Toguchi A.C., Lopes J.C., Pavione M.A., Enríquez S.K.T. (2024). Percutaneous closure of patent ductus arteriosus versus surgical treatment in low-birth-weight preterms: A systematic review and meta-analysis. Cardiol. Young.

[43] Wheeler C.R., Gagner D., Stephens H., Kraus A., Zurakowski D., Friedman K.G., Ibla J.C., Callahan R., Porras D., Levy P.T. (2022). Phenotyping respiratory decompensation following definitive closure of the patent ductus arteriosus in preterm infants. J. Perinatol..

[44] Clyman R., Cassady G., Kirklin J.K., Collins M., Philips J.B. (2009). The role of patent ductus arteriosus ligation in bronchopulmonary dysplasia: Reexamining a randomized controlled trial. J. Pediatr..

[45] Georgiev S., Tanase D., Eicken A., Hörer J., Zahn E., Borgmann K., Renner D., Ewert P. (2024). Mobile bedside ductus arteriosus closure in severely premature neonates using only echocardiographic guidance. Catheter. Cardiovasc. Interv..

[46] Mendel B., Kohar K., Djiu R.J., Yumnanisha D.A., Vidya A.P., Winarta J., Arifin K.H., Mumtaz M.D.E., Nugroho A.K.Z., Jagannatha G.N.P. (2025). Safety and Efficacy of Zero Fluoroscopy Patent Ductus Arteriosus Closure in Comparison to the Standardized Fluoroscopy-Guided Procedure: A Systematic Review and Meta-Analysis. Curr. Cardiol. Rev..

[47] Wang C., Zhang F., Ouyang W., Zhao G., Lu W., Zou M., Pan X. (2020). Transcatheter Closure of Patent Ductus Arteriosus under Echocardiography Guidance: A Randomized Controlled Noninferiority Trial. J. Interv. Cardiol..

